# Genetically predicted N-Acetyl-L-Alanine mediates the association between CD3 on activated and secreting Tregs and Guillain-Barre syndrome

**DOI:** 10.3389/fnins.2024.1398653

**Published:** 2024-09-20

**Authors:** Qi Lyu, Lianlian Zhang, Yasuo Ding, Zehao Liu

**Affiliations:** ^1^Department of Ultrasound, The Affiliated Taizhou People’s Hospital of Nanjing Medical University, Taizhou School of Clinical Medicine, Nanjing Medical University, Taizhou, China; ^2^Department of Ultrasonography, The Yancheng Clinical College of Xuzhou Medical University, The First People’s Hospital of Yancheng, Yancheng, China; ^3^Department of Neurosurgery, The Affiliated Taizhou People’s Hospital of Nanjing Medical University, Taizhou School of Clinical Medicine, Nanjing Medical University, Taizhou, China

**Keywords:** Guillain-Barre Syndrome, mendelian randomization analysis, immune cells traits, N-acetyl-L-alanine, plasma metabolites

## Abstract

**Objective:**

This study sought to explore the potential causal relationships among immune cell traits, Guillain-Barre syndrome (GBS) and metabolites.

**Methods:**

Employing a two-sample Mendelian randomization (MR) approach, the study investigated the causal associations between 731 immune cell traits, 1400 metabolite levels and GBS leveraging summary-level data from a genome-wide association study (GWAS). To ensure the reliability of our findings, we further assessed horizontal pleiotropy and heterogeneity and evaluated the stability of MR results using the Leave-one-out method.

**Results:**

This study revealed a causal relationship between CD3 on activated & secreting Tregs and GBS. Higher CD3 on activated and secreting Regulatory Tregs increased the risk of GBS (primary MR analysis odds ratio (OR) 1.31/SD increase, 95% confidence interval (CI) 1.08–1.58, *p* = 0.005). There was no reverse causality for GBS on CD3 on activated & secreting Tregs (*p* = 0.36). Plasma metabolite N-Acetyl-L-Alanine (ALA) was significantly positively correlated with GBS by using the IVW method (OR = 2.04, 95% CI, 1.26–3.30; *p* = 0.00038). CD3 on activated & secreting Tregs was found to be positively associated with ALA risk (IVW method, OR, 1.04; [95% CI, 1.01–1.07], *p* = 0.0078). Mediation MR analysis indicated the mediated proportion of CD3 on activated & secreting Tregs mediated by ALA was 10% (95%CI 2.63%, 17.4%).

**Conclusion:**

In conclusion, our study identified a causal relationship between the level of CD3 on activated & secreting Tregs and GBS by genetic means, with a considerable proportion of the effect mediated by ALA. In clinical practice, thus providing guidance for future clinical research.

## Introduction

Guillain-Barré syndrome (GBS) is characterized as an acute inflammatory disorder affecting the peripheral nervous system (PNS) (Israeli et al., 2012), presenting with initial weakness and sensory symptoms in the lower extremities that may progress to involve the upper limbs and cranial muscles (Shahrizaila et al., 2021). This infection-triggered condition, although uncommon, carries a potential for fatality, with a median incidence rate of approximately 1–2 cases per 100,000 person-years (Doets et al., 2022). Despite the heterogeneous clinical manifestations and various distinct clinical subtypes of GBS, a subset of patients, approximately 20%, may rapidly develop respiratory failure (Leonhard et al., 2019).

The inflammation of the peripheral nervous system is a significant factor in the pathophysiology of GBS, marked by a multifaceted inflammatory process affecting the demyelination and axonal integrity of peripheral nerves (Zheng et al., 2019; Blum and McCombe, 2014). Dysregulation of the immune system may impede the repair mechanisms of the peripheral nervous system, leading to sustained inflammation and contributing to the varied clinical presentations observed in GBS (Leonhard et al., 2022). Current research indicates a substantial body of evidence supporting the role of genetic factors in the development of this immune-mediated inflammatory disorder (Safa et al., 2021). Multiple studies have shown a correlation between GBS and hereditary neuropathies, suggesting that genetic abnormalities in peripheral neurons may heighten vulnerability to inflammatory reactions and the onset of GBS (Barreiro and Quintana-Murci, 2010; Dekker et al., 2009). Furthermore, familial clustering of GBS cases has been documented in several studies (Geleijns et al., 2004), and genetic susceptibility loci for GBS have been identified through genome-wide association studies (GWAS) and candidate gene analyses (Blum et al., 2018). Moreover, several studies have documented abnormal gene expression, specifically in genes related to immune function, in individuals with GBS (Liu et al., 2016; Zhang et al., 2018). Various polymorphisms in genes involved in immune response regulation have also been linked to GBS in diverse populations (Doets et al., 2018; Jaramillo-Valverde et al., 2019). The presence of subset-specific immune cells, characterized by unique combinations of surface proteins, further complicates the understanding of GBS pathogenesis (Olson et al., 2023; Súkeníková et al., 2024). However, limited research has investigated the potential causal relationships between GBS susceptibility and immune cell characteristics, including levels of cell surface antigens and relative/absolute counts of specific cell types. GWAS have discovered numerous genetic signals linked to immune-related diseases (Blum et al., 2018), yet the precise mechanisms and particular immune cell subtypes implicated in GBS remain largely unexplored. The causal relationship between specific immune cell characteristics and GBS has yet to be fully understood.

Cellular metabolism is integral to the human immune response against pathogenic infections (Yang L. et al., 2023; Yan et al., 2022). The buildup of certain metabolites can act as epigenetic regulators for immune cells, influencing the epigenetic profile of key metabolic enzymes (Bader et al., 2020). There is currently limited understanding regarding the mechanisms by which inflammation-induced alterations in host metabolites may disrupt immune system regulation in patients with GBS. To address this knowledge gap, the present study utilized a Mendelian randomization (MR) approach to examine the potential causal relationship between immune cell traits and GBS risk. Furthermore, associations were identified between GBS risk and blood metabolite levels, suggesting the existence of a potential mediator that could elucidate the mechanistic link between genetic variations in immune cell traits and disease susceptibility.

## Materials and methods

### Study design

All the data utilized in this investigation were obtained from publicly accessible publications, ethical approval and informed consent were obtained in the original research. Using a two-sample MR approach, we investigated the causal relationship between 731 immune cell traits and GBS using single nucleotide polymorphisms (SNPs). To demonstrate credible causality in MR, instrumental variables (IVs) must exhibit a strong association with the exposure, mitigate confounding between exposure and outcome, and influence the outcome.

### GWAS data sources for immune cell traits, GBS and ALA

From the GWAS Catalog, we extracted summary statistics for each immune trait (GCST900001391–GCST90002121), enveloping 731 immune cell traits (Orrù et al., 2020), all pertaining to the European demographic. These traits included absolute cell counts (AC) (*n* = 118), relative cell counts (RC) (*n* = 192), median fluorescence intensity (MFI) (*n* = 389), and morphological parameters (MP) (*n* = 32). Within the database, cell traits were classified as mature B cell, dendritic cell, mature T cell, myeloid, TBNK (T, B, and NK cells), and Treg panels.

For GBS, data labeled “Guillain-Barre syndrome” (finn-b-G6_GUILBAR) were used. This study encompassed 215,931 European individuals and 16,380,463 SNPs, all of which pertain to the European population, 213 were GBS cases, and 215,718 were controls.^1^

Summary statistics on N-acetyl-L-alanine (ALA) levels were hailing from a 2023 study (Chen et al., 2023) including 8,220 individuals all associated with the European population.

Data labeled GCST90200303.^2^

### Instrumental variable (IV) selection and data harmonization

A strict inclusion criteria was implemented to ensure the accuracy and effectiveness of establishing the causal relationship between immune cells and Guillain-Barré Syndrome (GBS). Instrumental variables (IVs) with a *P*-value below 5 × 10^–8^ were chosen for analysis (Wang et al., 2024). Additionally, SNPs exhibiting linkage disequilibrium were filtered out using criteria of Furthermore, SNPs demonstrating linkage disequilibrium were excluded based on the criteria of R^2^ < 0.001 and kb = 10000 to uphold the independence of the chosen instrumental variables and reduce the potential bias introduced by linkage disequilibrium effects. Eligible SNPs for exposure matching must satisfy the specified *P*-value threshold and be devoid of any linkage disequilibrium (Burgess and Thompson, 2011).

Moreover, in order to avoid bias from weak instrumental variables, we used the F-statistic to assess the statistical strength of the correlation between each SNP and the exposures. An IV with an F-statistic greater than 10 was considered a strong instrument, whereas one with an F-statistic less than 10 was considered weak. Essential details such as the effective allele and effective size (comprising β value, standard error, and P value) of each SNP are extracted for the computation of the F statistic to assess potential bias from weak instrumental variables (IVs). SNPs with palindromic structures were automatically excluded from each analysis. The F-statistic was calculated using the formula F = [(*N*–K–1)/K]/[R^2^/(1–R^2^)], where N represents the sample size of the exposure GWAS, K is the number of SNPs, *R*^2^ is the proportion of variance explained by the SNPs in the exposure database, SNPs with an *F*-statistic value less than 10 were excluded, since an F-statistic value greater than 10 indicates sufficient strength to ensure validity.

### Statistical analysis

Based on GWAS data, multiple MR methods were used to investigate the causal association between 731 immune traits, 1400 metabolite levels and GBS. Inverse-variance weighting (IVW) was primarily used, supplemented by the weighted median method and MR-Egger to ensure stability. The IVW method is widely utilized in Mendelian randomization research, necessitating that single nucleotide polymorphisms (SNPs) adhere to the three fundamental principles of MR Research to achieve accurate causal estimation (Yavorska and Burgess, 2017). The primary distinction between IVW and MR-Egger lies in the consideration of the intercept term in the regression analysis. In the presence of pleiotropy, results obtained through the Mendelian Randomization-Egger (MR-Egger) method are given precedence (Bowden et al., 2016; Bowden et al., 2015). In the presence of heterogeneity, the Weighted Median approach is preferred (Bowden et al., 2016; Bowden et al., 2015). If the IVW results were significant (*p* < 0.05) and aligned in trend with other methods, we regarded them as indicative of significance. Several sensitivity analyses were conducted to further validate our results. These included a heterogeneity assessment using Cochran’s Q statistic, horizontal pleiotropy detection via the intercept in MR-Egger regression and MR-Pleiotropy Residual Sum and Outlier (MR-PRESSO), as well as a leave-one-out analysis to evaluate the influence of individual SNPs. All data processing and statistical analyses were conducted in the R software (version 4.3.2), utilizing the TwoSampleMR (version 0.5.7) and MR-PRESSO (version 1.0) packages.

### Primary analysis

**FIGURE 1:**
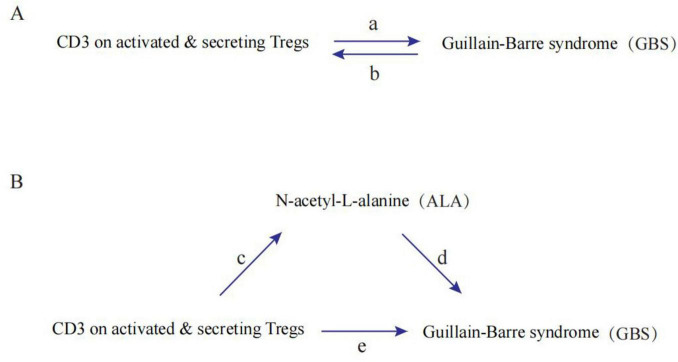
Diagrams illustrating associations examined in this study. **(A)** The total effect between CD3 on activated and secreting Tregs and Guillain-Barre syndrome (GBS). a is the total effect using genetically predicted CD3 on activated and secreting Tregs as exposure and GBS as outcome. b is the total effect using genetically predicted GBS as exposure and CD3 on activated and secreting Tregs as outcome. **(B)** The total effect was decomposed into: (1) indirect effect using a two-step approach (where c is the total effect of CD3 on activated and secreting Tregs on N-acetyl-L-alanine (ALA), and d is the effect of ALA on GBS) and the product method (c × d) and (2) direct effect (e = a–c × d). Proportion mediated was the indirect effect divided by the total effect.

Figure 1 shows a schematic summary of the analysis. We conducted a two-sample bidirectional MR to evaluate the mutual causality between CD3 on activated & secreting Tregs and GBS (Figure 1A), which was designated as the total effect. Meta-analysis is used to combine Wald ratios for causal effects for each SNP using inverse variance weighting (IVW). Additionally, MR-Egger and weighted-median were used to complement IVW. A variety of methods were used to estimate MR according to a variety of validity assumptions. It is based on the premise that all SNPs can be used as instrumental variables when applying IVW. As a result, this method provides accurate estimations. With MR-Egger, directional pleiotropy is assessed for instrumental variables, where the intercept is interpreted as an estimate of genetic pleiotropy. When compared to the MR-Egger analysis, the weighted median maintains higher precision (lower standard deviation). When horizontal pleiotropy is present, the weighted median can still offer a reliable estimate even when half of the genetic variants are not valid instrumental variables.

### Mediation analysis

A two-step MR design was used to conduct a mediation analysis investigating the role of ALA in mediating the causal pathway from CD3 on activated and secreting Tregs to GBS outcome (Figure 1B). The total impact can be broken down into an indirect impact (via mediators) and a direct impact (without mediators). The total effect of CD3 on activated and secreting Tregs on GBS was decomposed into direct effects of CD3 on activated & secreting Tregs on GBS (e in Figure 1B) and indirect effects mediated by CD3 on activated & secreting Tregs through the mediator (c × d in Figure 1B). We calculated the percentage mediated by the mediating effect by dividing the indirect effect by the total effect. Meanwhile, 95% confidence intervals were calculated with the delta method.

### Sensitivity analysis

MR Steiger filtering was used to test the causal direction of each SNP extracted in relation to exposure and outcome (Hemani et al., 2017). This technique computes the explained variance in exposure using instrumental SNPs and assesses if the variance in the results is lower than the exposure. “TRUE” MR Steiger results indicate causality in the expected direction, while “FALSE” results indicate causality in the opposite direction. We excluded SNPs with ‘FALSE’ results, indicating that it showed evidence of a major effect on the outcome rather than exposure. Heterogeneity between SNPs was assessed using Cochran’s Q statistic and funnel plots. Horizontal pleiotropy was detected using the MR-Egger intercept method. Finally, leave one-out analysis was used to validate the effect of each SNP on the overall causal estimates (Yuan et al., 2022).

## Results

### Association of CD3 on activated and secreting Tregs with GBS

**FIGURE 2:**
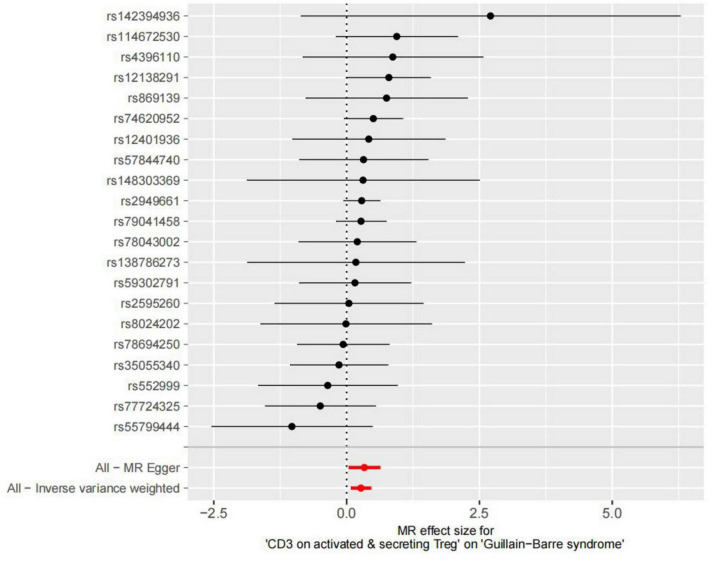
Forest plot to visualize causal effect of each single SNP on total GBS risk.

After removing palindromic and ambiguous SNPs, SNPs without proxy and SNPs with wrong causal directions identified by MR Steiger filtering, there were 21 SNPs in CD3 on activated & secreting Tregs as instrumental variables (Supplementary Table 1 and Figure 2).

IVW, MR-Egger, and weighted median regression were used to estimate the causal relationship between genetically predicted CD3 on activated & secreting Tregs and GBS. Across all three MR methods, there was broad and consistent support for the positive association of CD3 on activated & secreting Tregs with GBS (IVW odds ratio [OR] per SD increase in GBS = 1.31 [95% CI, 1.08–1.58], *p* = 0.005; MR-Egger OR per SD increase in GBS = 1.39 [95% CI, 1.03–1.87], *p* = 0.039; weighted median OR per SD increase in GBS = 1.32 [95% CI, 1.01–1.74], *p* = 0.044).

To rigorously control for confounding factors and avoid potential influence of GBS on immune cells, we did not include immune cells with a causal relationship in the reverse MR for GBS. The results of our MR analysis showed no reverse causality for GBS on CD3 on activated & secreting Tregs (*p* = 0.36).

### Association of CD3 on activated & secreting Tregs with ALA

**FIGURE 3:**
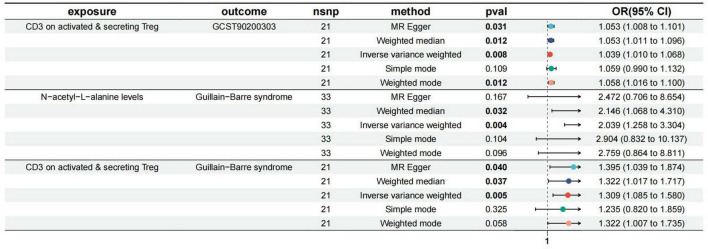
Forest plot to visualize the causal effects of N-acetyl-L-alanine (ALA) with CD3 on activated and secreting Tregs and GBS.

We extracted a total of 21 genome-wide significant SNPs as instrumental variables after removing palindromic and ambiguous SNPs, SNPs without proxies, and SNPs in the

wrong causal direction identified by MR Steiger filtering (Supplementary Table 2). According to the IVW, MR–Egger and weighted median methods, genetically predicted CD3 on activated & secreting Tregs was found to be positively associated with ALA risk (IVW method, OR, 1.04; [95% CI, 1.01–1.07], *p* = 0.0078; MR-Egger method, OR, 1.05; [95% CI, 1.00–1.10], *p* = 0.031; weighted median method, OR, 1.05; [95% CI, 1.01–1.10], *p* = 0.012). The results are shown in Figure 3.

### Association of ALA with GBS

As shown in Supplementary Table 3, we presented all genetic instruments associated with ALA at the genome-wide significance level (*p* < 5 × 10^–8^). As shown in Figure 3, genetically predicted ALA was significantly positively correlated with GBS [OR = 2.04, 95% CI, 1.26–3.30; *p* = 0.00038] by using the IVW method. Weighted median OR per SD increase in GBS = 2.15 [95% CI, 1.10–4.17], *p* = 0.024).

### Proportion of the association between CD3 on activated & secreting Tregs and GBS mediated by ALA

**FIGURE 4:**
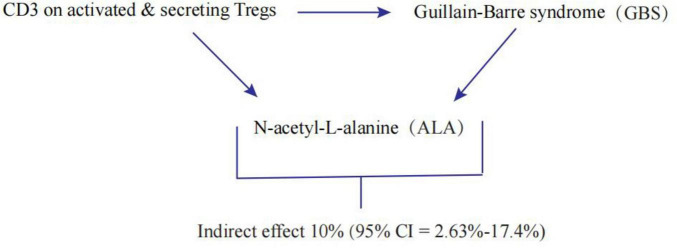
Schematic diagram of the N-acetyl-L-alanine (ALA) mediation effect.

We analyzed ALA as a mediator of the pathway from CD3 on activated & secreting Tregs to GBS. We found that CD3 on activated & secreting Tregs was associated with increased ALA, which in turn was associated with an increased risk of GBS. As shown in Figure 4, our study showed that ALA accounted for 10% of the increased risk of GBS associated with CD3 on activated & secreting Tregs (proportion mediated: 10%; 95% CI = 2.63%-17.4%).

### Analysis for heterogeneity and horizontal pleiotropy

To mitigate potential heterogeneity stemming from varying analysis platforms, experiments, populations, and other factors that may affect the results of MR analysis. We evaluated heterogeneity using both the IVW and MR-Egger test methods, with *P* < 0.05 indicating the presence of heterogeneity in the study. The results revealed that none of the intercepts from MR-Egger and IVW for the causal relationship between CD3 on activated & secreting Tregs, GBS and ALA were not statistically significant (*P* > 0.05), indicating that the findings remained unaffected by any potential bias stemming from heterogeneity (Supplementary Table 4). Additionally, funnel plots (Supplementary Figure 1) and scatter plots (Supplementary Figure 2) were also used to visually investigate heterogeneity, revealing no indication of SNPs impacting the overall causal relationship.

The MR-Egger intercepts for the causal relationship between CD3 on activated & secreting Tregs and GBS were not statistically significant (*P* = 0.589), suggesting an absence of genetic pleiotropy bias. The MR-Egger intercepts for the causal relationship between CD3 on activated & secreting Tregs and ALA were not statistically significant (*P* = 0.061). The MR-Egger intercepts for the causal relationship between GBS and ALS were not statistically significant (*P* = 0.745), affirming the robustness of the results.

The effect of each SNP on the overall causal estimates was verified by leave-one-out analysis (Supplementary Figure 3). After removing each SNP, we systematically performed the MR analysis again for the remaining SNPs. The results remained consistent, indicating that all SNPs were calculated to make the causal relationship significant.

## Discussion

This study utilized the two-sample Mendelian randomization method to investigate the causal relationship between 731 immune cell traits and the risk of Guillain-Barré Syndrome (GBS). The research represents a novel attempt to comprehensively elucidate the causal connection between immune phenotypes and GBS. By leveraging data from Genome-Wide Association Studies (GWASs), the study assessed 731 immune cell phenotypes and 1400 metabolites for their potential association with GBS. Our Mendelian randomization analysis identified CD3 as a marker on activated and secreting Tregs immune cell phenotypes that may impact the susceptibility to Guillain-Barré Syndrome (GBS). Among these phenotypes, N-acetyl-L-alanine (ALA) is hypothesized to play a role in the development of GBS as a potential mediator, with 10% of this effect being mediated through ALA.

Tregs are a subset of CD4+ T cells that exhibit anti-inflammatory properties, characterized by high levels of CD25 and the transcription factor forkhead box P3 (Foxp3) (Olson et al., 2023). These cells play a crucial role in maintaining immune balance and preventing autoimmunity by suppressing self-reactive T cells. Regulatory T cells (Tregs) have the ability to influence both innate and adaptive immunity by modulating various immune cell populations, including neutrophils, monocytes, antigen-presenting cells, B cells, and T cells (Zhang et al., 2019). Prior studies have elucidated the dysregulated functions of Treg cells in Guillain-Barré Syndrome (GBS), where chronic and excessive inflammation driven by effector T-cell-mediated immune responses can lead to neuronal cell death. The diminished quantity and proportion of Tregs have been correlated with elevated levels of proinflammatory Th1 and Th17 cytokines, coupled with a decrease in anti-inflammatory Th2 and Th3 cytokines, ultimately resulting in GBS (Zhang et al., 2013; Zepp et al., 2011; Li et al., 2014). The robust immunosuppressive function mediated by Tregs plays a crucial role in maintaining self-tolerance and preventing autoimmune diseases. However, dysfunction or inadequate functioning of this system can result in a state of heightened immune reactivity against self-antigens, leading to the destruction of host organs and the development of autoimmune diseases.

The presence of CD3 on activated and secreting Tregs has been shown to be significantly correlated with an elevated risk of Guillain-Barré Syndrome (GBS) in our study. Regulatory T cells (Tregs) play a critical role in modulating inflammatory responses, with their function being closely linked to the human leukocyte antigen (HLA) gene, which has been implicated in genetic studies of Guillain-Barré syndrome (GBS) (McCombe et al., 2006). The CD3 marker is universally present on the surface of T cells and serves as a vital component of the T cell receptor (TCR) complex, which is essential for T cell development and activation. The TCR specifically recognizes both endogenous and exogenous antigens, while CD3 facilitates T cell activation and proliferation by bridging the interaction between recognized antigens and effector signals (Shah et al., 2021). T cells play a central role in adaptive immunity by recognizing antigens presented by major histocompatibility complex (MHC) and T cell receptor (TCR) complexes. Our findings indicate that the presence of CD3 on activated and secreting regulatory T cells (Tregs) is positively correlated with Guillain-Barré Syndrome (GBS). The overexpression of CD3 is linked to prolonged antigen exposure. Activated and secreting Treg cells expressing CD3 are a subset of regulatory cells that modulate T cell responses both directly and indirectly. However, the precise role of CD3+ activated and secreting Treg subsets in Guillain-Barré Syndrome (GBS) remains incompletely understood. It is hypothesized that the activation state indicated by CD3 may be associated with a decreased frequency of Tregs, impairment of their suppressive function, and heightened reactivity and resistance to self-reactive effector T cell regulatory mechanisms.

ALA, a derivative of L-alanine, can be generated through the direct synthesis of N-acetyltransferases or the proteolytic breakdown of N-acetylated proteins by hydrolases, such as aminoacylase I (Perrier et al., 2005). The upregulation of N-acetyl-L-alanine (ALA) may indicate a reduction in non-acetylated L-alanine levels. Furthermore, ALA may potentially compete with non-acetylated L-alanine for binding to the same nutritional receptor, resulting in decreased L-alanine uptake and related outcomes. Yang (Yang B. et al., 2023) recently demonstrated that HIV-induced elevation of metabolites, specifically ALA, suppresses the production of pro-inflammatory cytokine IFN-γ by natural killer (NK) cells in individuals with latent tuberculosis infection (LTBI+). Additionally, ALA inhibits the glycolytic activity of NK cells from LTBI+ individuals in response to Mycobacterium tuberculosis (Mtb) infection. Cobre’s findings indicate that biomarkers, specifically N-Acetyl-l-Alanine, were found to be elevated in severe COVID-19 patients as opposed to individuals with mild COVID-19 or healthy controls (de Fátima Cobre et al., 2024). GBS is characterized by acute inflammatory polyradiculoneuropathy. Infections caused by bacteria or viruses can lead to substantial immunometabolic alterations in the host, potentially leading to competition for nutrients between the infecting agent and immune cells, thereby reducing the availability of glucose for infiltrating immune cells. Infection with a pathogen can lead to localized tissue hypoxia as a result of increased oxygen consumption by the invading bacteria (Sheppard et al., 2021). This acute inflammatory polyradiculoneuropathy also results in reduced glucose availability to immune cells in GBS, as glucose serves as a crucial energy source for bacteria. These physiological processes are closely linked to immune activation (Loftus and Finlay, 2016), as demonstrated by a previous study highlighting the importance of alanine in CD4+ T-cell activation.

In health, T lymphocytes are in a resting state. However, in disease states stimulation with their cognate antigen induces massive growth and proliferation. Ron-Harel et al. (2019) have demonstrated that T cell activation is dependent on extracellular alanine. The consumed alanine is primarily utilized for protein synthesis, and deprivation of alanine impairs T cell metabolism and effector functions. To directly investigate the catabolic fate of alanine, alanine tracing experiments were conducted. The results revealed that the only substantially 13C-labeled aqueous metabolites detected in these tracing experiments were alanine itself and its acetylated derivative, namely N-acetyl-L-alanine (ALA). Our study identified a causal relationship between the expression level of CD3 on activated and secreting T regulatory cells (Tregs) and GBS through genetic analysis. The activation and secretion of Tregs induced by various factors may impact the susceptibility of GBS. A significant portion of this effect was mediated by ALA, the catabolic substrate of alanine. Collectively, the immune-neuro-metabolic interactions form a closed loop in GBS. Consequently, tracing the catabolic fate of alanine in activated T cells may serve as a potential predictor of GBS progression and prognosis.

Various studies have identified genetic risk loci for Guillain-Barré syndrome (GBS) through genome-wide association studies (GWAS) or candidate gene approaches, highlighting the importance of gene expression, particularly those related to the immune infection. HLA alleles have been extensively studied in Guillain-Barré Syndrome (GBS) (Li et al., 2000; Bodis et al., 2018), as well as the relationship between GBS and immunoglobulin G Fc receptors (FcγRs). Subsequent research has explored the connection between GBS and polymorphisms in FcγRs and various other immune-related genes (Zhang et al., 2018; Wu et al., 2012). Additionally, single nucleotide polymorphisms (SNPs) within cytokine coding genes, specifically IL-17 and TNF-α, have been identified as potential risk factors for GBS (Li et al., 2012; Créange et al., 1996). There is a lack of systemic research investigating the potential causal relationship between immune cell traits and Guillain-Barré Syndrome (GBS). Recent genome-wide association studies (GWASs) have utilized flow cytometry to profile large-scale immune cell traits and have offered valuable resources for exploring such associations. Our study represents the first Mendelian randomization (MR) analysis to investigate the causal effects of complex genetic signatures in immune cells on GBS, as far as we are aware. MR leverages genetic variants as instrumental variables, held constant at GBS, to make causal inferences about the influence of modifiable risk factors, thereby potentially mitigating certain forms of confounding. Our study conducted a thorough analysis of the relationship between CD3 expression on activated and secreting regulatory T cells and Guillain-Barré Syndrome, with ALA potentially acting as a mediator. Our results demonstrate intricate genetic control of immune cells that have distinct effects on the susceptibility to autoimmune diseases at the level of cell subtypes. These findings highlight potential drug targets that could be utilized in the development of more precise therapies for autoimmune disorders.

This study is subject to several limitations. In our study, we utilized data labeled “Guillain-Barre syndrome” (finn-b-G6_GUILBAR). This dataset included 215,931 individuals and 16,380,463 single nucleotide polymorphisms (SNPs), all pertaining to the Finnish population. Firstly, the data on immune cells-related immune traits used in our GWAS meta-analysis were predominantly derived from the European population. As such, the generalizability of our results to non-European populations remains uncertain and warrants further investigation in future MR analyses. Secondly, the dataset did not provide any subcategory classifications. Consequently, the generalizability of our findings to different subtypes within the population remains uncertain and necessitates further investigation in future studies. Lastly, heterogeneous effects exist across individuals. The rarity of GBS results in a relatively small sample size for recruitment variables in our study. Consequently, it is imperative to expand the dataset and implement stricter inclusion criteria to enhance the validity of future research findings.

## Conclusion

In summary, this study provides a thorough analysis of causal estimates regarding the relationship between 731 immune cell traits and GBS, as well as the potential mediation through MR approaches. Our findings demonstrate a causal link between the expression levels of CD3 on activated and secreting Tregs and GBS through genetic mechanisms, with a significant portion of the effect being mediated by ALA. In a clinical setting, identifying the risk allele for GBS has practical implications for predicting individuals at risk for the condition, avoiding exposure to risk factors, and designing personalized therapeutic modalities.

## Data Availability

The datasets presented in this study can be found in online repositories. The names of the repository/repositories and accession number(s) can be found in the article/Supplementary material.

## Footnotes

1
https://gwas.mrcieu.ac.uk/datasets/finn-b-G6_GUILBAR/

2
http://ftp.ebi.ac.uk/pub/databases/gwas/summary_statistics/GCST90200001-GCST90201000/GCST90200303/GCST90200303_buildGRCh38.tsv.gz

## Supplementary material

Supplementary Figure 1 Funnel plots to visualize overall heterogeneity of MR estimates for the effect of CD3 on activated & secreting Tregs on GBS.

Supplementary Figure 2 Scatter plots to visualize overall heterogeneity of MR estimates for the effect of CD3 on activated & secreting Tregs on GBS.

Supplementary Figure 3 Leave-one-out plot to visualize causal effect of CD3 on activated & secreting Tregs on GBS risk when leaving one SNP out.
