# Supplementary figures and images for: Genetically predicted N-Acetyl-L-Alanine mediates the association between CD3 on activated and secreting Tregs and Guillain-Barre syndrome
Source: Front Neurosci. 2024 Sep 20;18:1398653. doi: 10.3389/fnins.2024.1398653 (PMC11450862; doi:10.3389/fnins.2024.1398653)

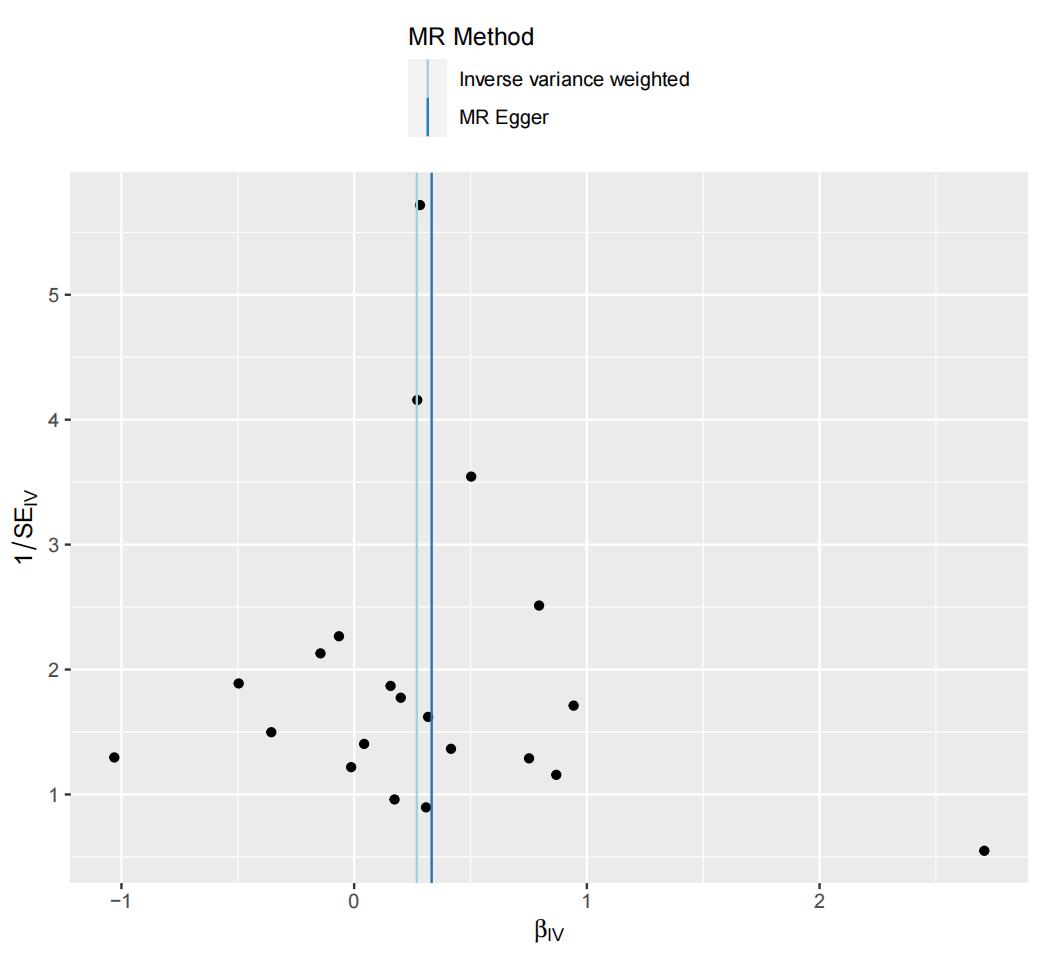

Supplement: Supplementary Figure 1 — Funnel plots to visualize overall heterogeneity of MR estimates for the effect of CD3 on activated & secreting Tregs on GBS. [file Image_1.jpg]

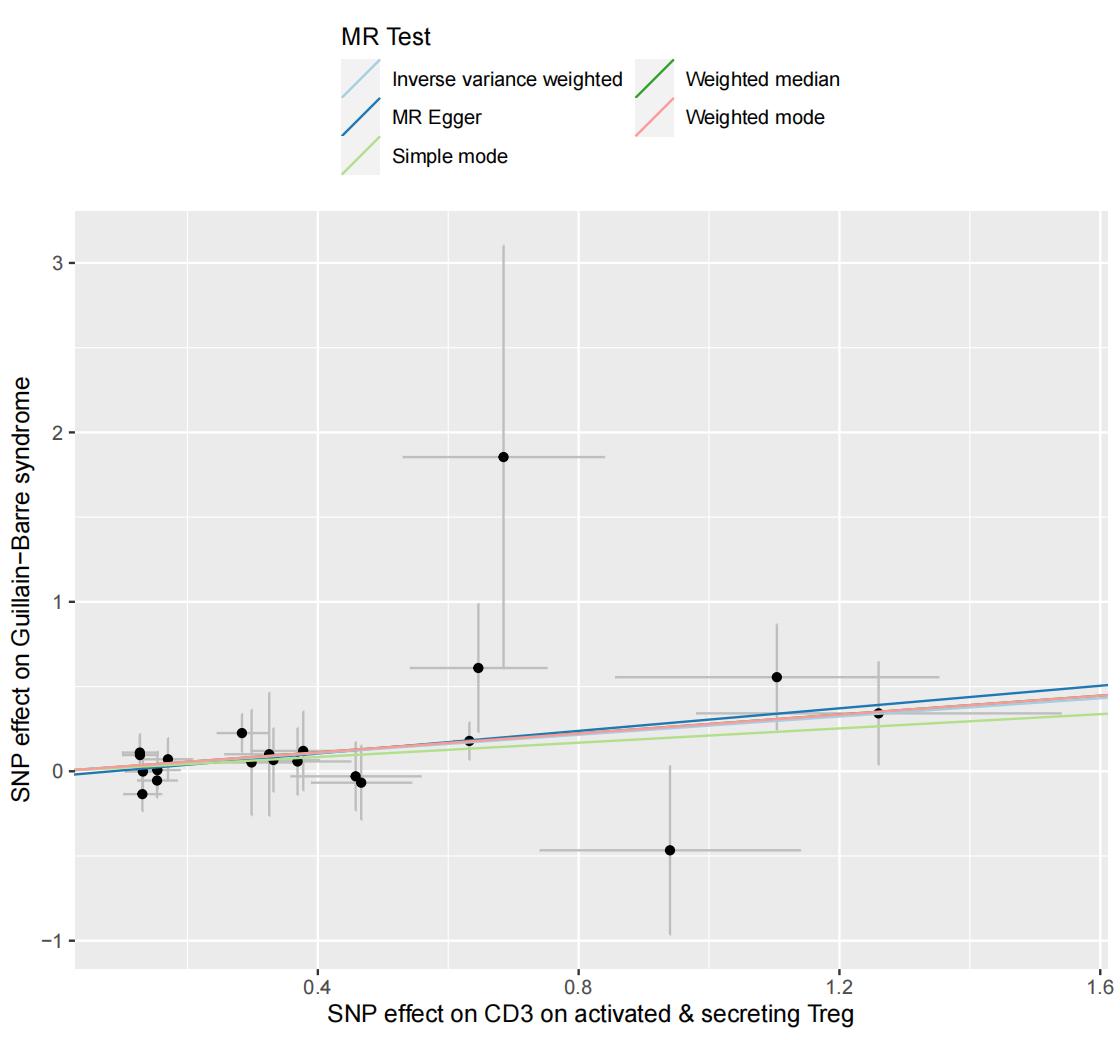

Supplement: Supplementary Figure 2 — Scatter plots to visualize overall heterogeneity of MR estimates for the effect of CD3 on activated & secreting Tregs on GBS. [file Image_2.jpg]

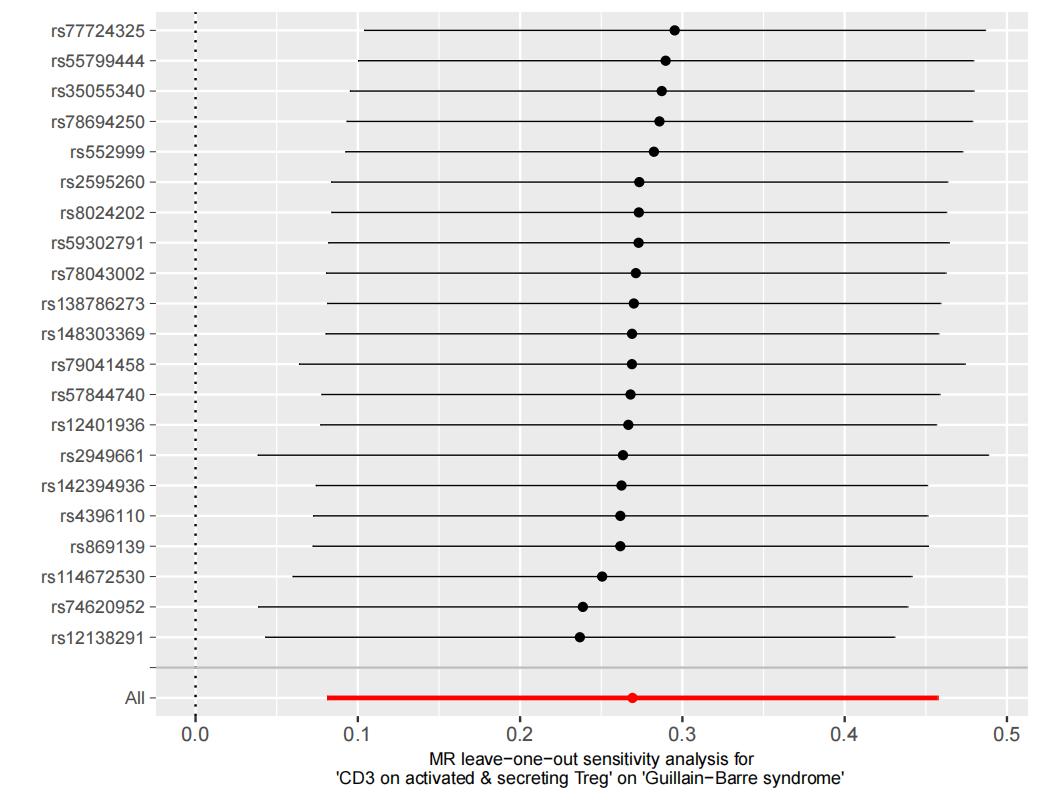

Supplement: Supplementary Figure 3 — Leave-one-out plot to visualize causal effect of CD3 on activated & secreting Tregs on GBS risk when leaving one SNP out. [file Image_3.jpg]
